# Toward the Era of a One-Stop Imaging Service Using an Angiography Suite for Neurovascular Disorders

**DOI:** 10.1155/2013/873614

**Published:** 2013-05-14

**Authors:** Sheng-Che Hung, Chung-Jung Lin, Wan-Yuo Guo, Feng-Chi Chang, Chao-Bao Luo, Michael Mu-Huo Teng, Cheng-Yen Chang

**Affiliations:** ^1^Department of Radiology, Taipei Veterans General Hospital, 201, Section 2, Shih-Pai Road, Taipei 112, Taiwan; ^2^National Yang-Ming University, Taipei 112, Taiwan

## Abstract

Transportation of patients requiring multiple diagnostic and imaging-guided therapeutic modalities is unavoidable in current radiological practice. This clinical scenario causes time delays and increased risk in the management of stroke and other neurovascular emergencies. Since the emergence of flat-detector technology in imaging practice in recent decades, studies have proven that flat-detector X-ray angiography in conjunction with contrast medium injection and specialized reconstruction algorithms can provide not only high-quality and high-resolution CT-like images but also functional information. This improvement in imaging technology allows quantitative assessment of intracranial hemodynamics and, subsequently in the same imaging session, provides treatment guidance for patients with neurovascular disorders by using only a flat-detector angiographic suite—a so-called one-stop quantitative imaging service (OSIS). In this paper, we review the recent developments in the field of flat-detector imaging and share our experience of applying this technology in neurovascular disorders such as acute ischemic stroke, cerebral aneurysm, and stenoocclusive carotid diseases.

## 1. An Actual Clinical Scenario of Stroke Management

A 67-year-old man presented at the hospital emergency unit with symptoms of acute right hemiplegia of less than 6 hours' duration. After a rapid assessment, the patient was taken to the computed tomography (CT) room, where noncontrast CT scan excluded intracranial hemorrhage. Immediate CT angiography depicted an occlusion at the proximal portion of the left middle cerebral artery, and a subsequent perfusion study identified a large penumbra, which manifested as prolonged time to peak (TTP) and preserved cerebral blood flow (CBF).

Because of the proximal cerebral artery occlusion and the risk of cell death in a large area of brain parenchyma, the patient was sent to the angiographic suite for revascularization with intra-arterial approach. After an interventional procedure lasting more than an hour, the occluded left middle cerebral artery was opened by mechanical thrombectomy and a few smaller occluded branches were left untreated. The patient was then sent back to the stroke intensive care unit. He was followed up on the next day by CT angiography and perfusion imaging using multidetector CT (MDCT). The studies showed normal hemodynamic parameters in most of the penumbra and no hemorrhage in the brain parenchyma. He was discharged a few days later under a favorable clinical status with only minor neurological deficits.

## 2. Introduction

Recently, angiographic suites equipped with flat detectors have become a standard imaging practice. Flat-detector imaging is also known as flat-detector computed tomography (FDCT) or angiographic CT. Remarkable advances in imaging technology over the recent years have resulted in notable improvement of imaging acquisition and postprocessing techniques in FDCT. In addition to digital subtraction angiography (DSA), which is obtained by subtraction of images before from after contrast medium injection and removing superimposed bone and soft tissue densities [1], FDCT can provide CT-like brain parenchyma images (DynaCT) and three-dimensional morphological and hemodynamic datasets of vasculatures, by combining one or more C-arm rotations. Consequently, a one-stop peritherapeutic imaging service has become feasible.

In this paper, we review the recent progress of FDCT technology in angiographic suite and share our clinical experience in coupling these imaging techniques with the clinical workflow of neurovascular disorders.

## 3. Technical Principles of Flat-Detector CT

A state-of-the-art flat panel detector consisted of two independent layers. The first is a fluorescence scintillator screen of cesium iodide, which absorbs and converts the X-rays into light photons, and the second is a layer of photodiodes, made up of hydrogenated amorphous silicon to convert light photons into a digital signal [2, 3]. The original development of flat-panel detectors was aimed at improving standard radiography by providing a higher dynamic range and a fast and repeated direct digital readout. With the improvement of three-dimensional reconstruction techniques [4–7] and reduction of artifacts, the concept of applying flat detectors for X-ray computed tomography had been investigated for several years. The FDCT can be installed in a gantry similar to that of a conventional MDCT [8], or on a C-arm system. The C-arm based system is named because of its configuration and used primarily for fluoroscopic imaging during surgical and angiographic procedures. It can be immobile, mounted on the floor or ceiling of the angiographic room, or mobile that can operate in any medical scenario, for example, operation theater or intensive care unit. In this paper, the term FDCT refers to an angiographic suite equipped with a C-arm system and a flat detector. The system can generate CT images by a series of projection data over an angular range of 180 degrees plus fan angle. If equipped with a large size detector to cover a wide field of view, for example, 40 by 40 cm^2^, the FDCT can obtain a large scanning volume in a single rotation. Thus, the term of cone beam FDCT is interchangeably used in the literature [9].

The earlier works of C-arm CT where originally performed by using an conventional image intensifier system in 1990s [10–12]. However, because of inherent limitations, namely, low dynamic range, image distortion, and low-contrast detectability, the application was limited to 3D rotational angiography that allowed visualizing high-contrast vessels typically by employing intra-arterial contrast medium injections [12, 13]. Compared with image intensifiers, flat detectors offer superior image quality, including improved detective quantum efficiency (DQE), modulation transfer function (MTF), dynamic range, and dose efficiency [14]. Furthermore, a C-arm system equipped with flat detectors is capable of providing projection radiography, fluoroscopy, DSA, and CT-like images in one imaging suite. Thus, the C-arm FDCT gains wide popularity in current imaging practice because of the versatile applications in the angiographic suite [15, 16].

## 4. Angiographic Suite Equipped with FDCT

An angiographic suite equipped with FDCT enables early recognition of intracranial complications during endovascular therapeutic procedures, such as coiling an intracranial aneurysm, stenting a stenotic artery, or other interventional procedures [17, 18]. With its high spatial imaging resolution and capacity for correcting metallic artifacts, DynaCT can detect most brain parenchymal hemorrhages in emergency situations [18, 19]. The combination of DSA with DynaCT has proven superior to two-dimensional or even three-dimensional DSA alone for the management of neuroendovascular complications [20].

In conjunction with intra-arterial or intravenous contrast medium injection, FDCT angiography provides images with higher spatial resolution than MDCT angiography does. Moreover, FDCT is able to directly demonstrate the relationship between endovascular devices or vascular malformations and the surrounding parenchymal structures for guiding a treatment or planning a therapeutic strategy. The direct demonstration has not been possible since the invention of conventional DSA early in the last century [21, 22].

## 5. Functional Imaging

For functional analysis of hemodynamics, the contrast medium injection protocol and imaging reconstruction algorithm of FDCT are modified and optimized. By subtracting a mask rotation run from a contrast medium-filled rotation run, in which contrast medium in tissues reaches a static state after bolus contrast medium injection, FDCT enables measurement of parenchymal cerebral blood volume (FDCT-PBV) [23, 24]. In a preliminary study by Struffert et al., the CBV values obtained with FDCT-PBV were strongly correlated to those obtained with MDCT. The mean difference of CBV values between FDCT and MDCT was small (0.04 ± 0.55 mL/100 mL) [25]. On-site CBV measurement enables peritherapeutic monitoring of hemodynamics and allows a timely management when endovascular treatments are encountered. 

Advancements in protocols of contrast medium injection, imaging acquisition, rotational speed, and reconstruction algorithms have improved remarkably the temporal resolution of C-arm FDCT for hemodynamic measurement. A recent study by Ganguly et al. demonstrated the feasibility of measuring CBF, CBV, and mean transit time (MTT) directly with C-arm FDCT. The hemodynamic maps generated from C-arm FDCT correlated well with CT perfusion maps from MDCT [26].

## 6. Clinical Experience

### 6.1. Acute Ischemic Stroke

According to the World Health Organization (WHO), stroke is the second leading cause of death worldwide (10.8%). Furthermore, human neural tissues are rapidly and irreversibly lost at an estimated rate of 1.9 million neurons each minute as stroke progresses [27]. These facts emphasize the importance of emergent management of stroke both from medical and public health aspects although patient's clinical status at presentation and appropriate treatments being given (medical, endovascular, and surgical approaches) determine the final clinical outcome. 

Clinically, the time interval between ictus and hospital arrival determines the treatment option in stroke management. Advancements in imaging technology and imaging expertise, however, play a role in modifying the treatment paradigm. Magnetic resonance imaging and CT provide morphological and functional data regarding brain tissues that correlate well with stroke in the temporal and spatial domains. This information forms the foundation for treatment options. The evolving clinical scenario toward earlier diagnosis, prompter revascularization of occluded vessels, more timely tissue salvage, and better neurological function preservation becomes clinically appealing. Consequently, FDCT, as a one-stop imaging technique, provides pretherapeutic anatomic and physiological information for diagnosis and minimizes the time interval between diagnosis and revascularization procedure when a subsequent interventional neurovascular procedure is dynamically needed. 

**Figure 1:**
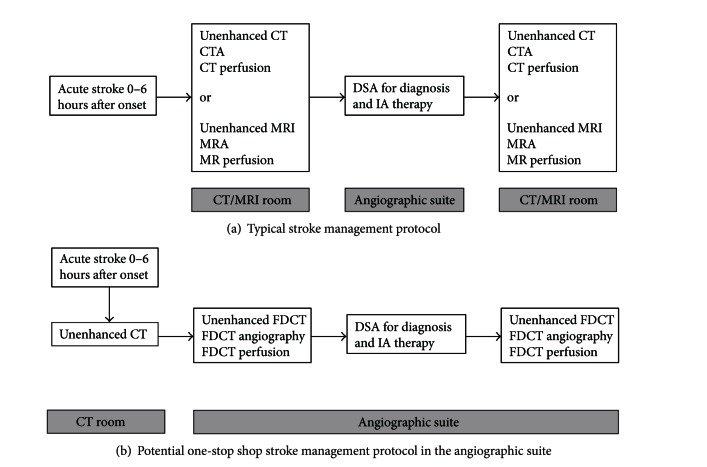
Shift of clinical paradigm in stroke management by employing flat-detector angiographic suite.

By combining C-arm rotational acquisition with intra-arterial contrast medium injection from the aortic arch, we can obtain (1) noncontrast DynaCT, (2) a three-dimensional volume of intracranial vasculature, and (3) an FDCT-PBV map. Noncontrast DynaCT helps detect intracranial hemorrhage at any peritherapeutic time point. DynaCT reliably detected intracerebral hematomas with an overall sensitivity up to 93.3% in a study of 44 patients [19], but lower when hematomas were small, located in the posterior fossa or adjacent to the skull base. DynaCT is also less sensitive to detect perimesencephalic subarachnoid hemorrhages (SAH) or minimal intraventricular hemorrhage. Peritherapeutic FDCT-PBV maps help identify the infarct core immediately before mechanical thrombectomy, guide the treatment decision, and predict final infarct size immediately following revascularization [28, 29] (Figure 1).

A partly hypothetical clinical scenario of stroke management of the same patient as reported in the first paragraph is as follows.

**Figure 2:**
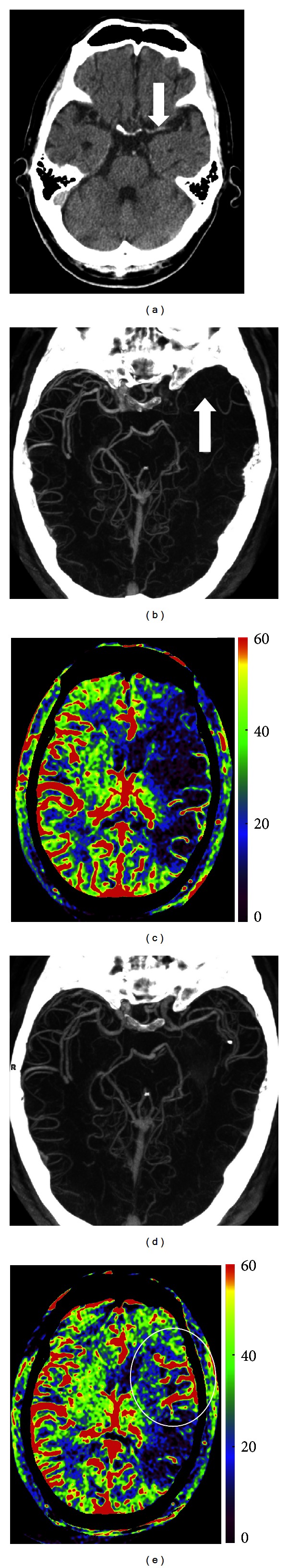
A 67-year-old man with left middle cerebral artery (MCA) occlusion. (a) Noncontrast computed tomography (CT) demonstrated a hyperdense MCA sign (arrow) and excluded intracranial hemorrhage. (b) Flat-detector CT (FDCT) angiography demonstrated the total occlusion of left MCA (arrow). (c) A parenchymal cerebral blood volume (FDCT-PBV) map depicted a large area of hypoperfusion in the corresponding left MCA territory, which was similar to the results of multidetector CT perfusion imaging (not shown). (d) After intra-arterial mechanical thrombectomy, recanalization of the left MCA was demonstrated by FDCT angiography. (e) An FDCT-PBV map depicted the recovery of CBV values (circle) in part of the hypoperfused parenchyma after revascularization.

A 67-year-old man arrives at the hospital emergency unit with symptoms of acute right hemiplegia of less than 6 hours' duration. After clinical evaluation and exclusion of intracranial hemorrhage by initial noncontrast MDCT, the patient is directly transferred to an FDCT angiographic suite for vascular and perfusion imaging, where preparation for the revascularization procedure starts simultaneously. FDCT angiography shows total occlusion of the left middle cerebral artery and FDCT-PBV demonstrates an area of hypoperfusion in the left frontoparietal lobes. An intra-arterial revascularization procedure by mechanical thrombectomy starts immediately. The occluded middle cerebral artery is opened in less than an hour. A few smaller occluded branches are left untreated. Immediately after revascularization, noncontrast DynaCT excludes intracranial hemorrhage. After the endovascular treatment, the patient is sent back to the stroke intensive care unit. Clinically, the posttherapeutic course is smooth. No imaging followup is requested. The patient is discharged a few days later with only minor neurological deficits and favorable clinical status (Figure 2).

### 6.2. Cerebral Aneurysm

Since the International Subarachnoid Aneurysm Trial (ISAT) and the Analysis of Treatment by Endovascular Approach of Nonruptured Aneurysms (ATENA), endovascular treatment has been established as a first-line treatment in the management of ruptured and nonruptured aneurysms [30, 31].

**Figure 3:**
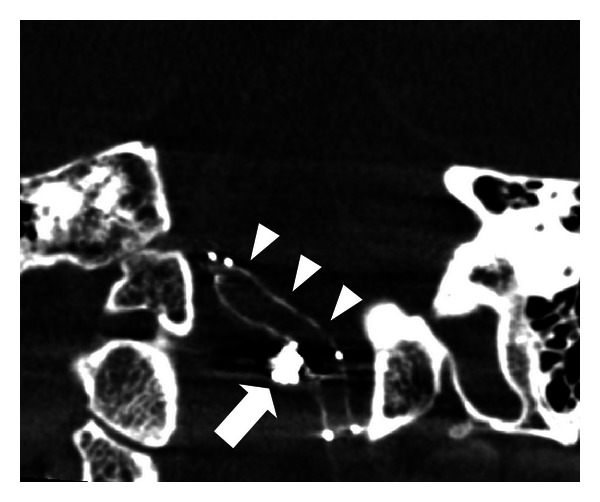
Intraprocedural DynaCT showed the relationship between stent struts (arrowheads) and coil mass (arrow) of a 50-year-old woman who received stent-assisted embolization for a left posterior inferior cerebellar artery aneurysm.

In our practice, DynaCT can be used to evaluate the degree of hydrocephalus and visualize the position of a shunt [32], monitor the extent of SAH immediately before and after endovascular procedures, and exclude intracranial complications before transferring the patient back to the ward from an angiographic suite [33]. For patients with broad-based cerebral aneurysms requiring stent-assisted coil embolization or flow diverter stents, precise confirmation of their positioning and relationship is important for endovascular treatment. However, stent struts are not radiopaque, and only the proximal and distal radiopaque stent markers are visible in fluoroscopy. Fluoroscopic localization and detection of stent morphology are difficult after deployment. Intraprocedural DynaCT enhances visualization of stent positioning with high spatial and contrast resolution and clearly illustrates the relationship between stent struts and coil mass (Figure 3). In a cohort study of eleven patients undergoing stent-assisted aneurysm embolization, the stent visibility was excellent in small aneurysms. However, the visibility was deteriorated in aneurysms larger than 10 mm in diameter due to beam hardening artifacts [34]. Besides, DynaCT enables early recognition of procedure-related complications, such as incomplete stent deployment, stent migration, stent fracture, and coil dislocation [35–37].

One of the future applications of the one-stop angiography suite is management of cerebral vasospasm. Cerebral vasospasm with delayed cerebral ischemia is the leading cause of morbidity and mortality in patients with aneurysmal SAH and survived from the initial hemorrhage. DSA is used as the reference standard, but not all angiographic vasospasms are clinically symptomatic. A combination of CT angiography and CT perfusion is reported to yield high diagnostic accuracy and can potentially improve the diagnosis of cerebral vasospasm after SAH [38]. Our preliminary experience shows that FDCT-PBV maps may help to identify severely ischemic brain parenchyma. By reformatting the sources images of FDCT-PBV, we can detect mild or moderate stenosis in proximal cerebral arteries with sensitivities of 84.7% and 90% by two independent raters, respectively, in a series of consecutive ten exams. This may optimize the treatment protocol of chemical angioplasty, which involves intra-arterial infusion of nimodipine, a dihydropyridine calcium channel blocker.

### 6.3. Stenoocclusive Carotid Disease

**Figure 4:**
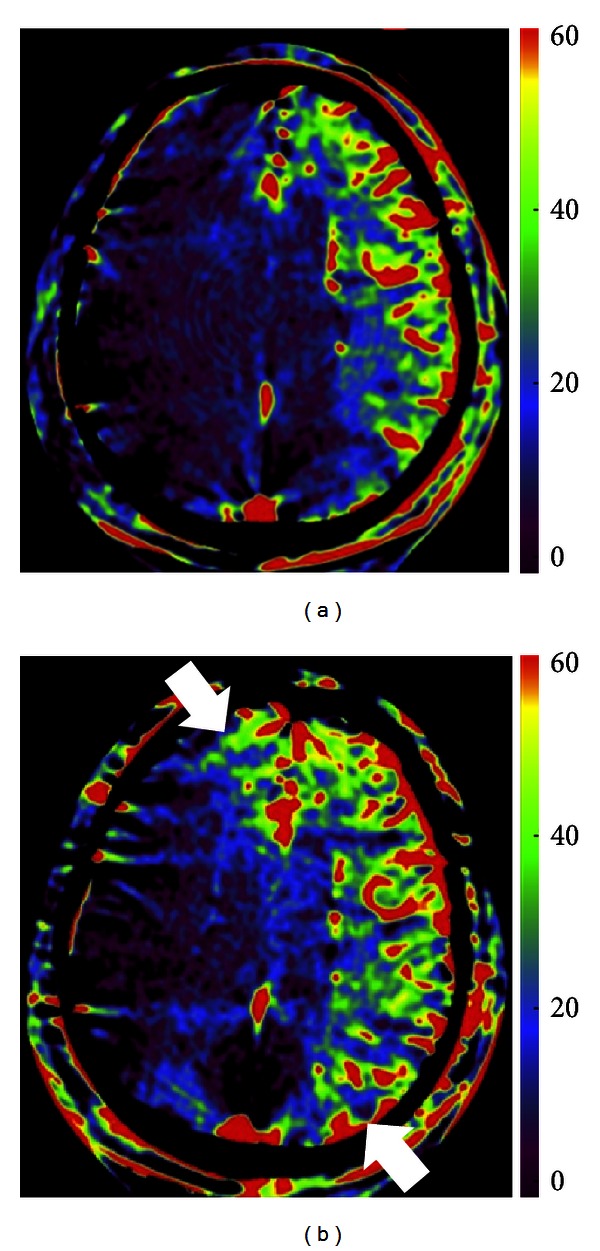
A 77-year-old man who underwent carotid artery stenting for left internal carotid artery high-grade stenosis. Prestenting (a) and poststenting (b) parenchymal cerebral blood volume (FDCT-PBV) maps in conjunction with selective intra-arterial contrast medium injection demonstrated increased CBV values and arterial territorial shifting (arrows).

In symptomatic intracranial atherosclerotic disease, intracranial angioplasty and stenting are increasingly used as a therapeutic option [39–42]. Similar to the situation in stent-assisted coil embolization of intracranial aneurysms, DynaCT and FDCT angiography are capable of visualizing the strut of a stent *per se* and the surroundings during the procedure [37, 43]. Moreover, the peritherapeutic FDCT-PBV maps demonstrate hemodynamic improvements in CBV values and arterial territory shifting (Figure 4). We found that peritherapeutic CBV changes were inconsistent and variable, which concurred with previous studies [44, 45].

With the high incidence of in-stent restenosis (8%–30%) [46–48], regular imaging followup is mandatory for patients who have undergone intracranial stenting for their stenoocclusive arteries. DSA, although invasive, is the standard follow-up imaging. FDCT angiography with intravenous contrast medium injection is less invasive than transarterial DSA and provides superior spatial resolution compared with MDCT angiography in both parenchymal and vascular imaging [49].

## 7. Conclusion

In conclusion, FDCT angiographic suite provides one-stop imaging for neurovascular disorders with the following advantages.

### 7.1. On-Site Assessment of Peritherapeutic Intracranial Conditions

Intracranial conditions, for example, ventricular size and hemorrhage, may occur or change after emergent treatments or deteriorate between/during the procedures of endovascular treatment. Moreover, the hemodynamic status and infarct core may dynamically evolve along the time course of stroke. The freedom of reassessing brain morphology and hemodynamics at any time during the endovascular procedure of stroke treatment provides a precise and updated intracranial roadmap for timely tailoring of treatment plan.

### 7.2. Prompt Initiation of Intra-Arterial Revascularization Treatment

Selected patients, for example, those with suspected acute ischemic stroke (<6 hours) or suspected post-SAH vasospasm, could be brought directly to an FDCT angiographic suite where DynaCT, FDCT angiography, and FDCT-PBV could be obtained with one stop. The imaging service algorithm could optimize the overall workflow of stroke management by avoiding patient relocation among imaging scanners and data transferal. The new paradigm might minimize the risk and time delay of interventional procedures.
